# National Confederation of State Medical Examining and Licensing Boards

**Published:** 1910-08

**Authors:** George H. Matson

**Affiliations:** Secretary; Columbus, Ohio


### National Confederation of State Medical Examining and Licensing Boards

REPORTED BY GEORGE H. MATSON, M.D., Secretary, Columbus, Ohio.

THE twentieth annual convention of the National Confederation of State Medical Examining and Licensing Boards was called to order at the Southern Hotel, St. Louis, by the president, Dr. A. Ravogli, of Cincinnati, June 6, 1910. After an invocation by the Reverend Stephen F. Sherman, Jr., Dr. A. H. Hamel, Chairman of the local Committee of Arrangements, introduced Dr. Joseph S. Grindon who delivered the address of welcome, which was responded to by Dr. Joseph C. Guernsey, vice-president, of Philadelphia.

A. Ravogli delivered the annual address of the president, entitled "A Plea for More Practical and Extended Clinical Instruction for Medical Students." The report of the secretary-treasurer, Dr. M. G. Motter, was read, audited and approved, showing a total of $557.89 with a balance of $228.08. The report of the Executive Council, read by Dr. N. R. Coleman, was referred to a committee which commended the subject matter for its practical value to medical educators, and recommended the adoption of an amendment to the constitution providing for a corresponding secretary, and the appointment of a committee to confer with a committee from the American Confederation of Reciprocating Examining and Licensing Medical Boards on the feasibility of uniting the two bodies. According to the subsequent report of this committee, the proposed union was found to be impracticable at present. The report of the committee on clinical instruction, by Dr. Henry Beates, Jr., and that on Materia Medica, by Dr. M. G. Motter, were read and referred for publication.

A paper was read by Dr. Abraham Flexner, of the Carnegie Foundation, on "The State Boards in Relation to Medical Education." This paper elicited a lively discussion and was the subject of considerable newspaper comment.

The symposium on clinical instruction was opened by Dr. George Dock, who said that present school conditions warrant the introduction by the boards of practical examinations, that the material is available, that their purpose should be to test the applicants' methods and that this would necessitate expert examiners. Dr. Fred C. Zapffe contributed a valuable and timely paper on the present status of clinical instruction. Dr. J. C. Guernsey discussed the advantages of the old preceptor system; Dr. J. C. Oliver opposed the lengthening of the medical course to five years; Dr. A. F. Stephens dwelt upon the importance of post-graduate clinical work. With reference to the introduction of practical examinations by the State Boards, Dr. Thomas McCrae discussed instruction in clinical medicine; Dr. A. D. Bevan, instruction in clinical surgery; Dr. C. F. Hoover, instruction in diseases of the heart and lungs; Dr. W. A. Hardaway, diseases of the skin; and Dr. D. T. Vail, refraction. Dr. J. Coons outlined the methods of practical examinations in histology, pathology, bacteriology, urinalysis, and other topics.

The Board of Medical Examiners of Utah, the State Medical Board of Arkansas Medical Society, the Eclectic State Medical Board of Arkansas, and Drs. E. J. Collins, A. H. Hamel, Frederic Singer, Darlington Snyder, R. O. Tucker, and N. P. Colwell were admitted to membership in the confederation.

The following officers and committees were elected: president, J. C. Guernsey, Philadelphia; first vice-president, James A. Egan, Springfield, Ill.; 2d vice-president, Charles A. Tuttle, New Haven Conn.; secretary-treasurer, George H. Matson, Columbus, O.; assistant secretary, Darlington Snyder, Columbus, O.; executive council, N. R. Coleman, Columbus, O.; Edwin B. Harvey, Boston; James A. Duncan, Toledo; A. H. Hamel, St. Louis; D. P. Madoux, Chester, Pa. Committee on clinical instruction, Henry Beates, Jr., Philadelphia; Charles A. Tuttle, New Haven, Conn.; Fred C. Zapffe, Chicago; Maurice J. Lewi, New York; L. F. Bennett, Beloit, Wis. Committee on materia medica, M. G. Motter, Washington, D. C.; J. C. Guernsey, Philadelphia; George MacDonald, Washington, D. C. Committee on lay publicity, Darlington Snyder, Columbus, O.; Frederic Singer, Pueblo, Col.; Fred C. Zapffe, Chicago. Committee on Abraham Flexner's paper: W. J. Means, N. P. Colwell and Henry Beates, Jr.

